# Native Medical Practice in India

**Published:** 1887-09-03

**Authors:** 


### Native Medical Practice in India.

By a Major-General of the Bengal Army.

During a protracted service in the Bengal Presidency I had many opportunities of conversing with the "Hakims," or native physicians, and observing their mode of treatment. No doubt these Indian doctors are quite ignored by the Fellows of the College of Physicians, and are probably regarded as "elderly gentlemen who have a good many old women's cures." I have been what is called a practical man all my life, and have tried to gain information from any and every source. I am one who does not think "that the nineteenth century lives in the sunshine, but that every other century spent its days in the coal-cellar." I have the greatest faith in the "pill and physic-bottle," when used so as to produce beneficial results: and my appreciation of results is not diminished when they are brought about by men who have never seen the inside of an European hospital. The first case I will record is that of a coolie boy who suffered from an enlarged spleen. I consulted a native Hakim. He told me to procure a quarter of a bottle of common gin, and into this he put borax—about the size of a walnut. Of this mixture he directed me to give the youth one dessert-spoonful every morning, early, before he took any food whatever; and twice more during the day, in a wine-glass of water. I did so, and in three or four days the spleen was down.

On another occasion a travelling Hakim came to my house and offered to sell a cure for ringworm. I had a native servant covered with ringworm, and the Hakim said he would guarantee a cure. He applied his remedy, and cured the servant in a few days. I then said to him, "Now, I will give you five rupees to tell me how this stuff is made." He then told me to send to the bazaar for some "Tūlsi" (holy-basil—*Ocymum sanctum*). Then he ground it and added some "Turma" or antimony; then some goat's milk. These, when compounded, he made up into oval balls the size of large marbles, and let them dry. After that he took a common stone and ground some of this down into a paste with water that had stood all night, and applied it. This was now just like ground slate-pencil in colour. I brought several of these balls home and gave them to my own doctor in London. He used them all, and asked me for more. He assured me that in his hands this remedy cured ringworm in three or four days. The Hindoos adore this holy-basil, and they probably did so originally for some good reason. I commanded a native regiment in Bengal for sixteen years, and the Sepoys used to grow the holy-basil just at the entrance of their lines or barrack-huts. I believe it is used to prevent the effects of malaria, as the sunflower is; the latter, which probably has some of the properties of the gum-tree (eucalyptus), is planted in marshy districts, and is a febrifuge. If the dry sunflower be rubbed just before the seeds get hard, there is given off an aroma similar to that of the leaves of the eucalyptus.

I once saw a man with a sore finger, like what is called "house-maid's thumb" in England. On this a native servant put a lemon with a hole in it as a poultice. The whitlow soon got well. The lemon should be applied before pus has formed. Perhaps the acid prevents the formation of pus.

For tape-worm the natives give the root of the pomegranate, and, if the root is not procurable, the pounded rind. The latter is, however, not so effective as the former. For intestinal worms the fruit of the mulberry is given to children. This is a very simple remedy. The native Ayahs always recommend it, and I have often found it beneficial.

On one occasion a female servant of ours was stung by a scorpion. The other Ayah said, "Get some lime, unslaked." I did so, and put it on, mixed with brandy or whiskey. This created intense heat, but the pain left almost immediately. The very next night the other Ayah was stung by another scorpion, and I again applied the lime and brandy, with the same satisfactory result.

For kidney affections the Ayahs cut up a large onion into slices, and pour hot water on it. They give a teaspoonful of this at a time to children.

For cholera the Sikhs take small cupfuls of onion uice made in this way. They regard it as an excellent remedy. It acts on the kidneys, and seems to be of decided service. I have seen this treatment used in many hundreds of cases, and apparently with great benefit.

